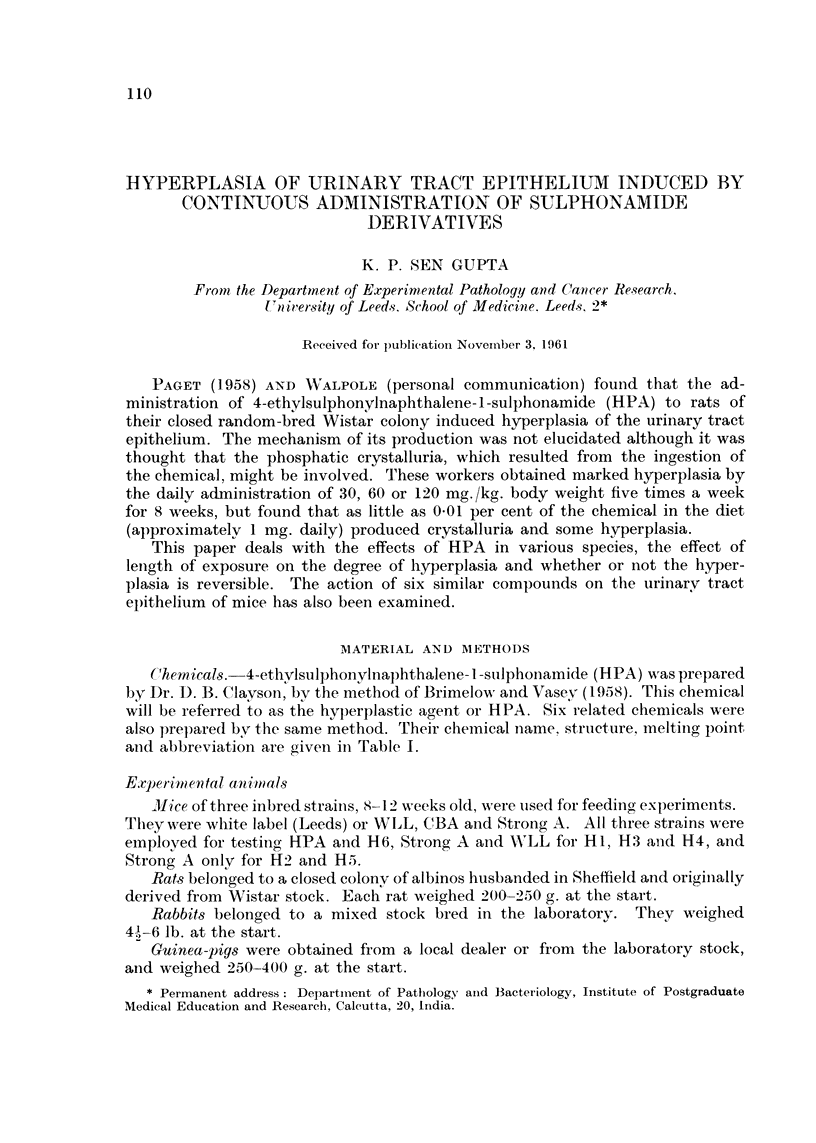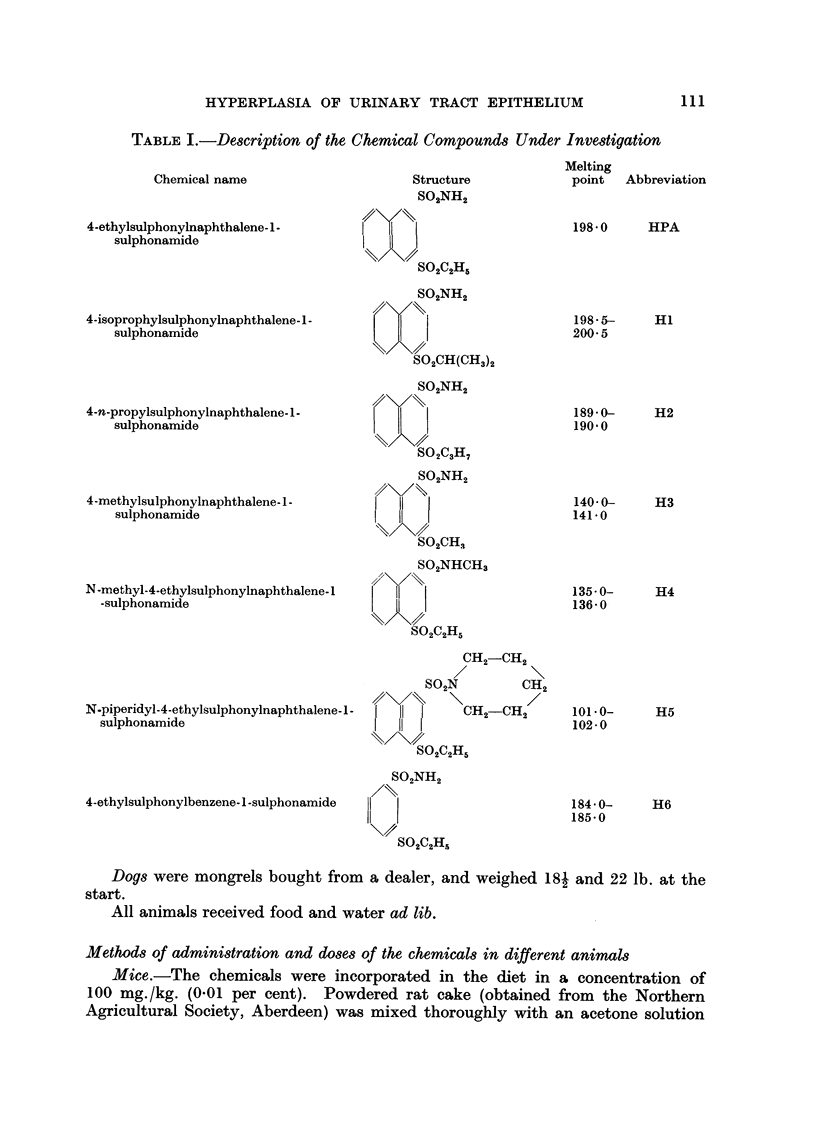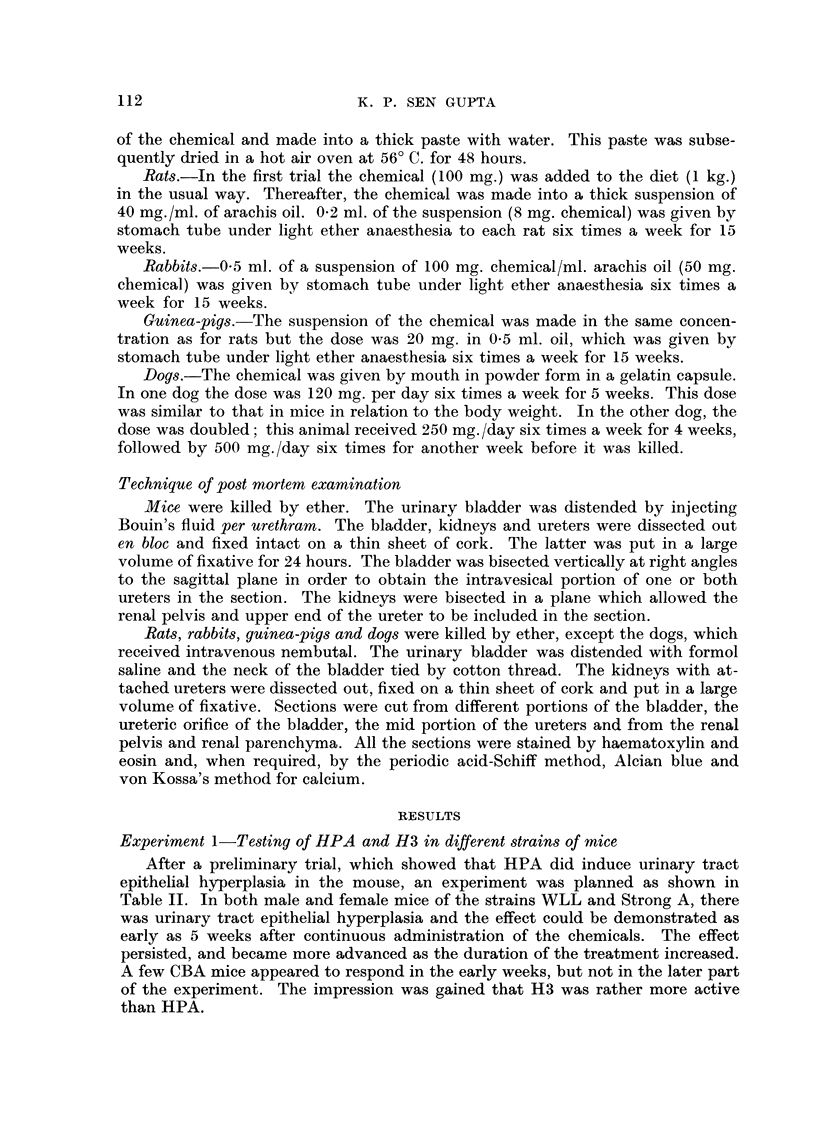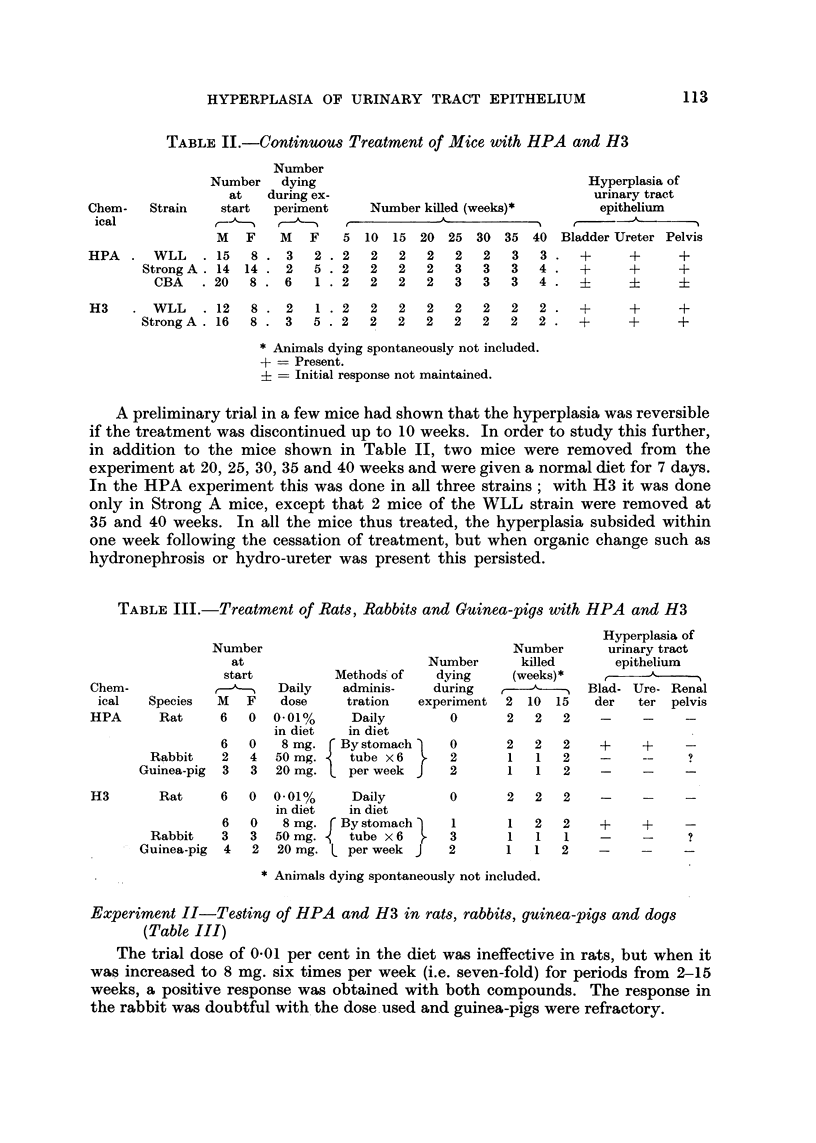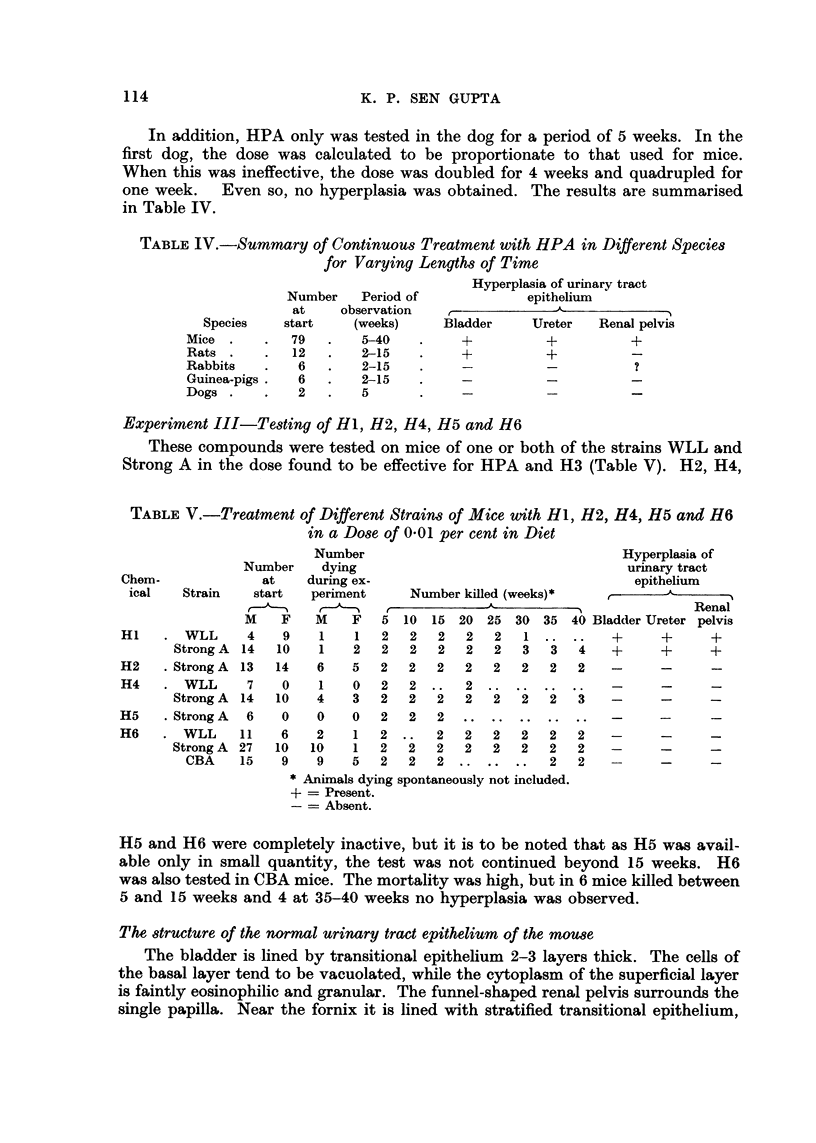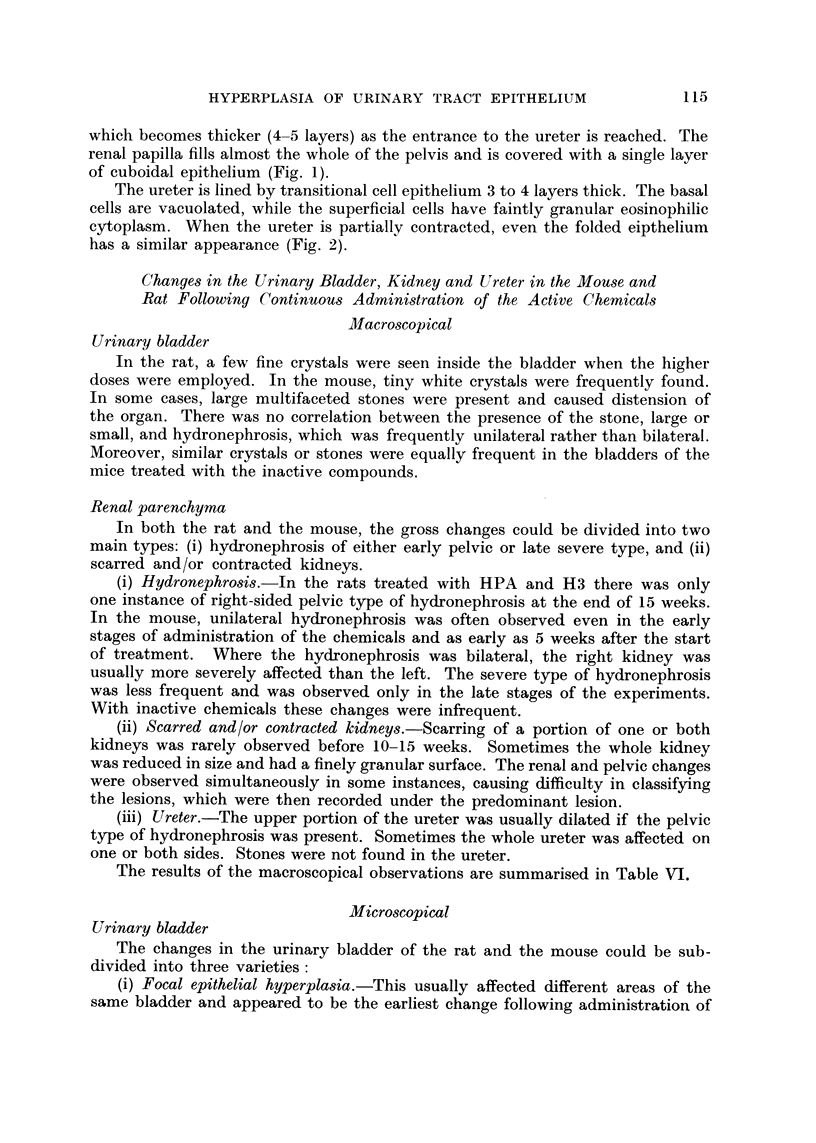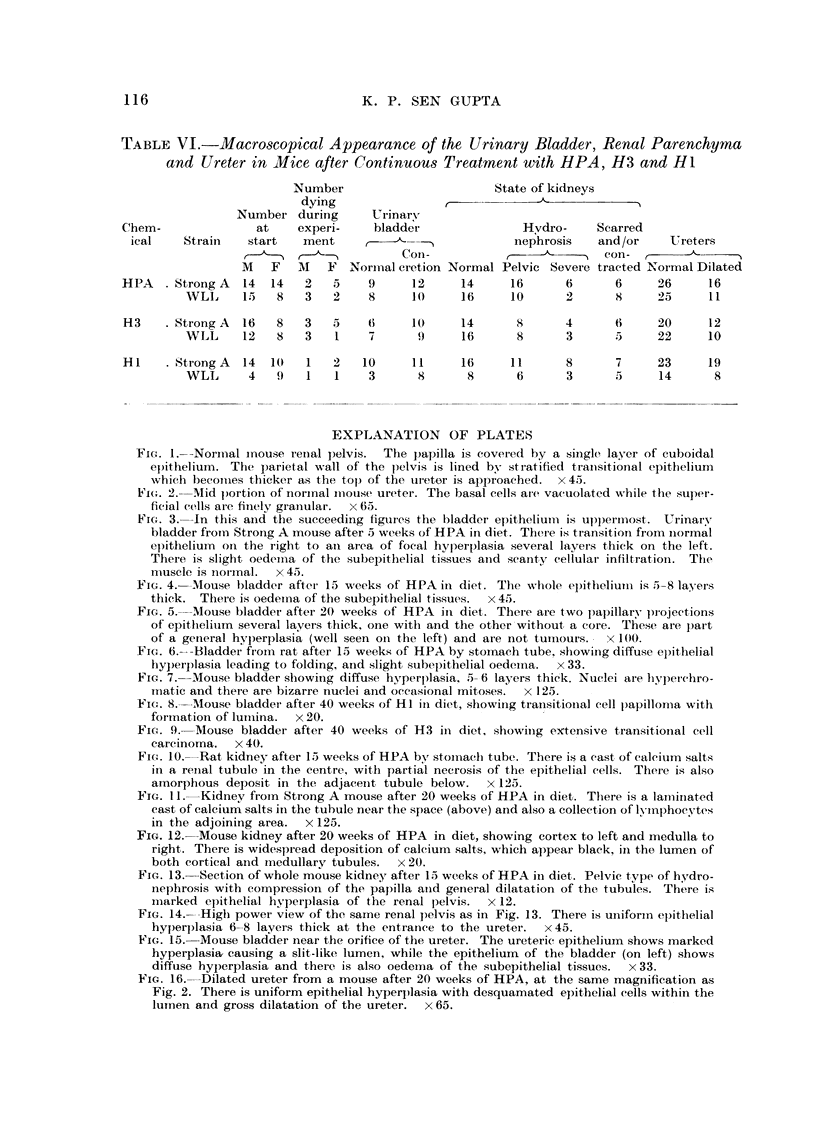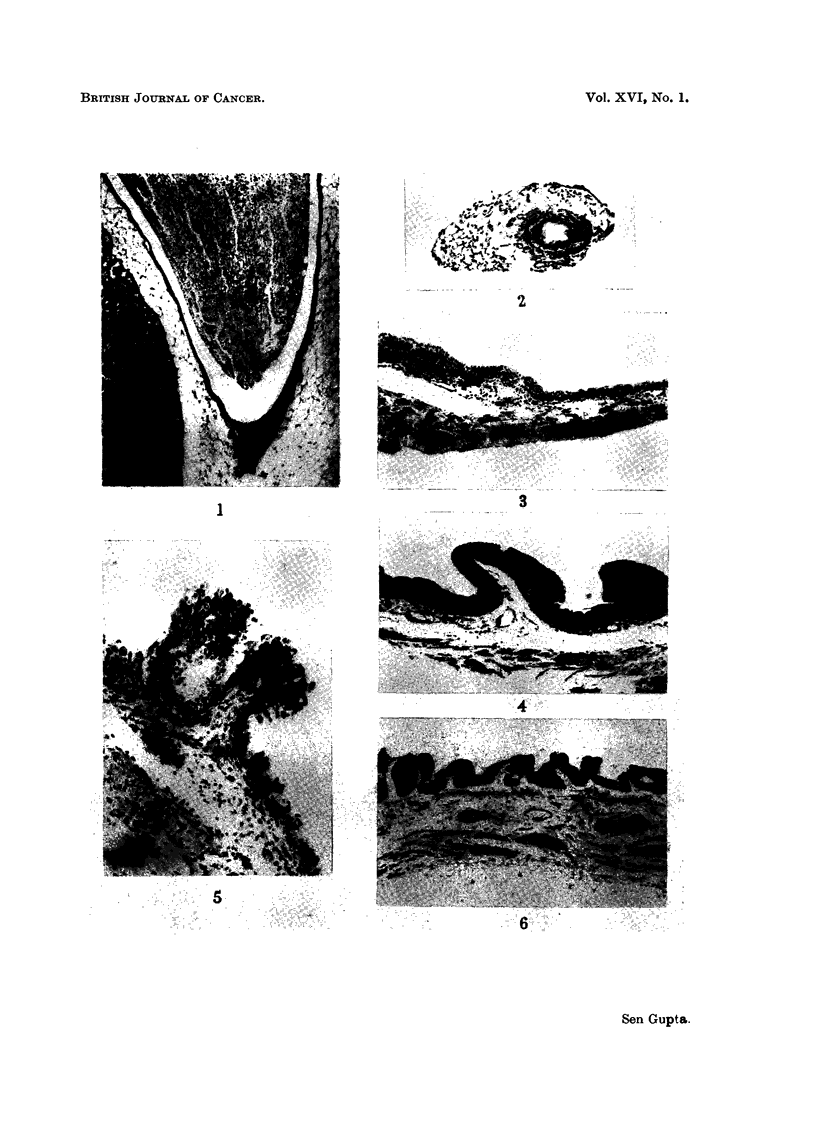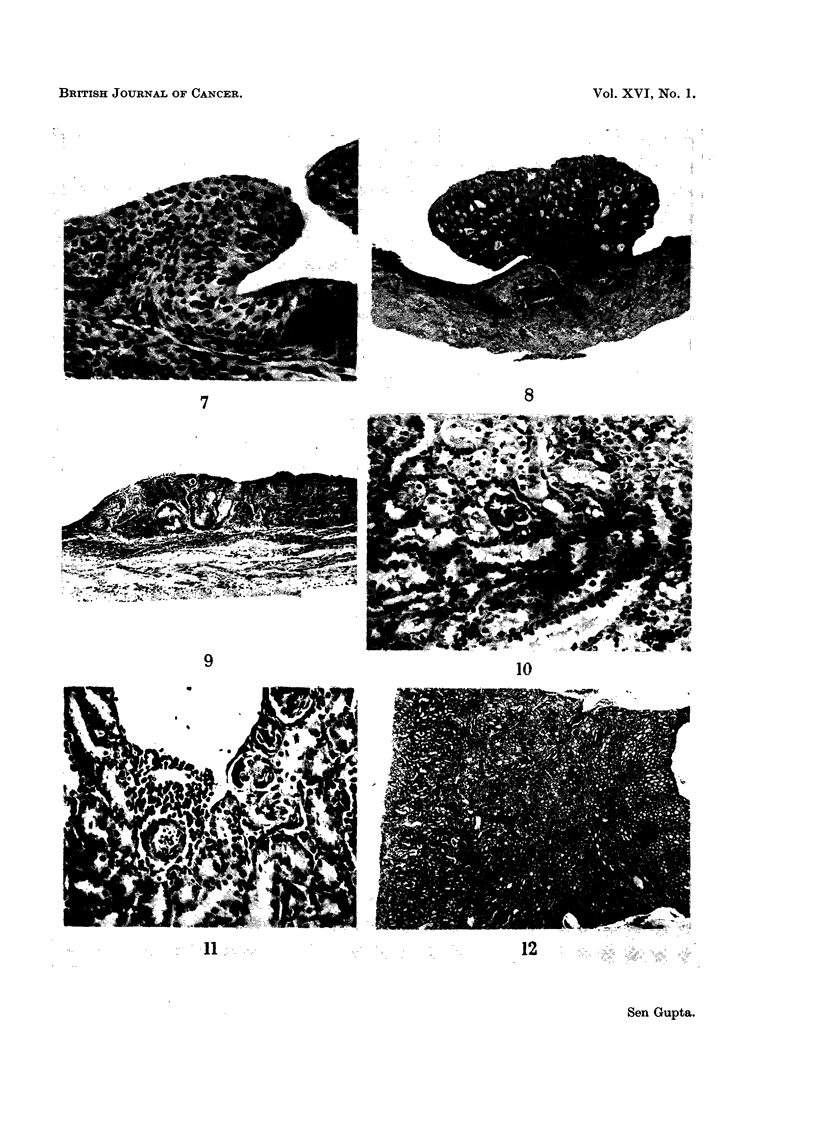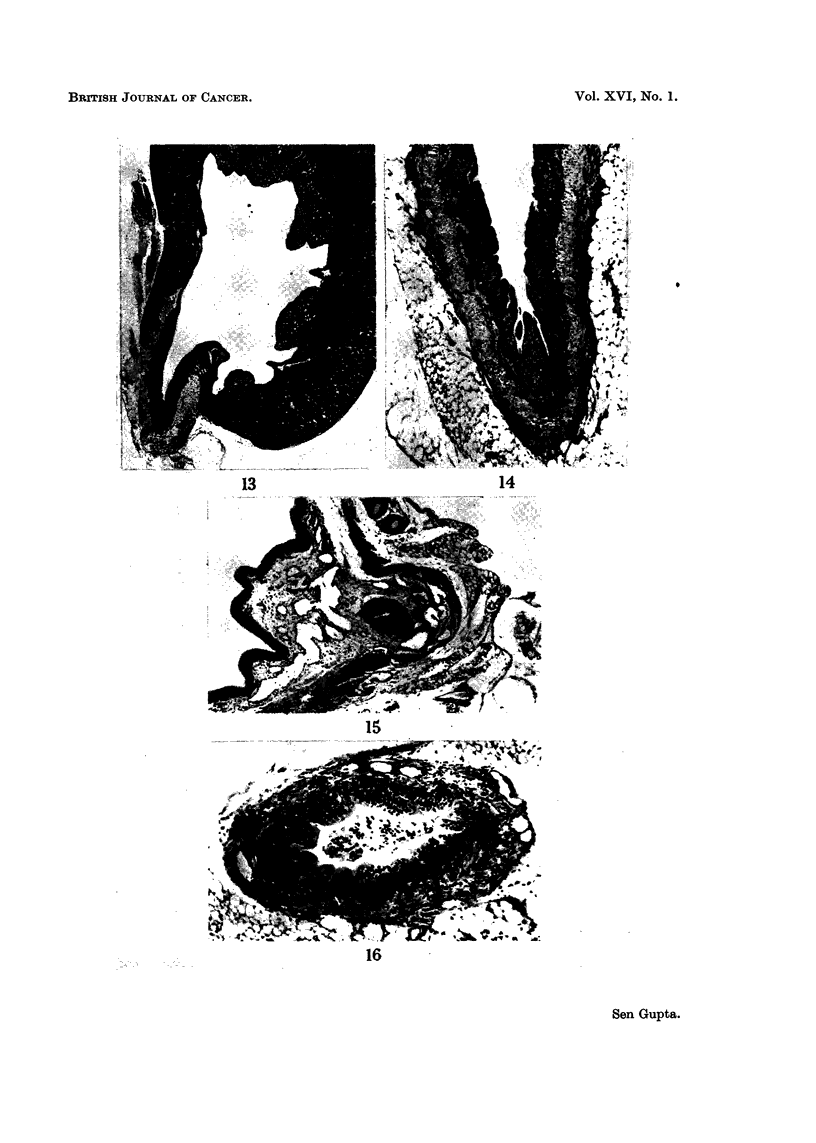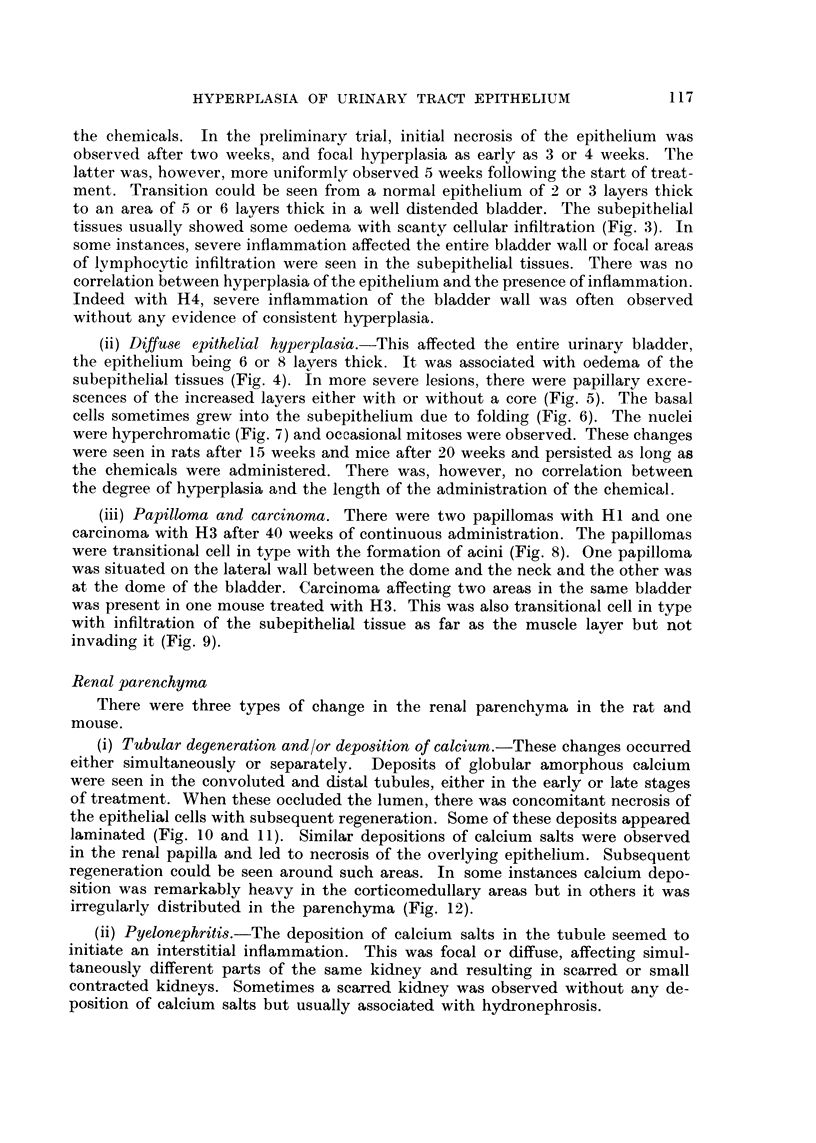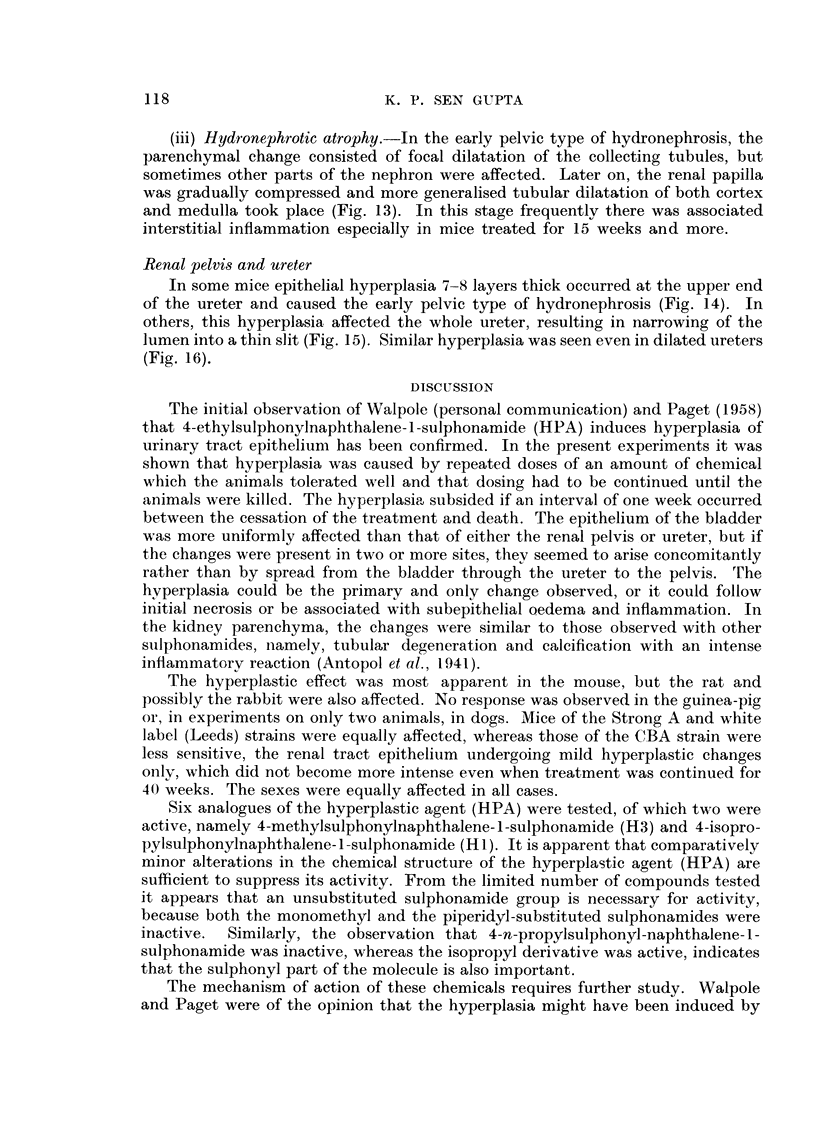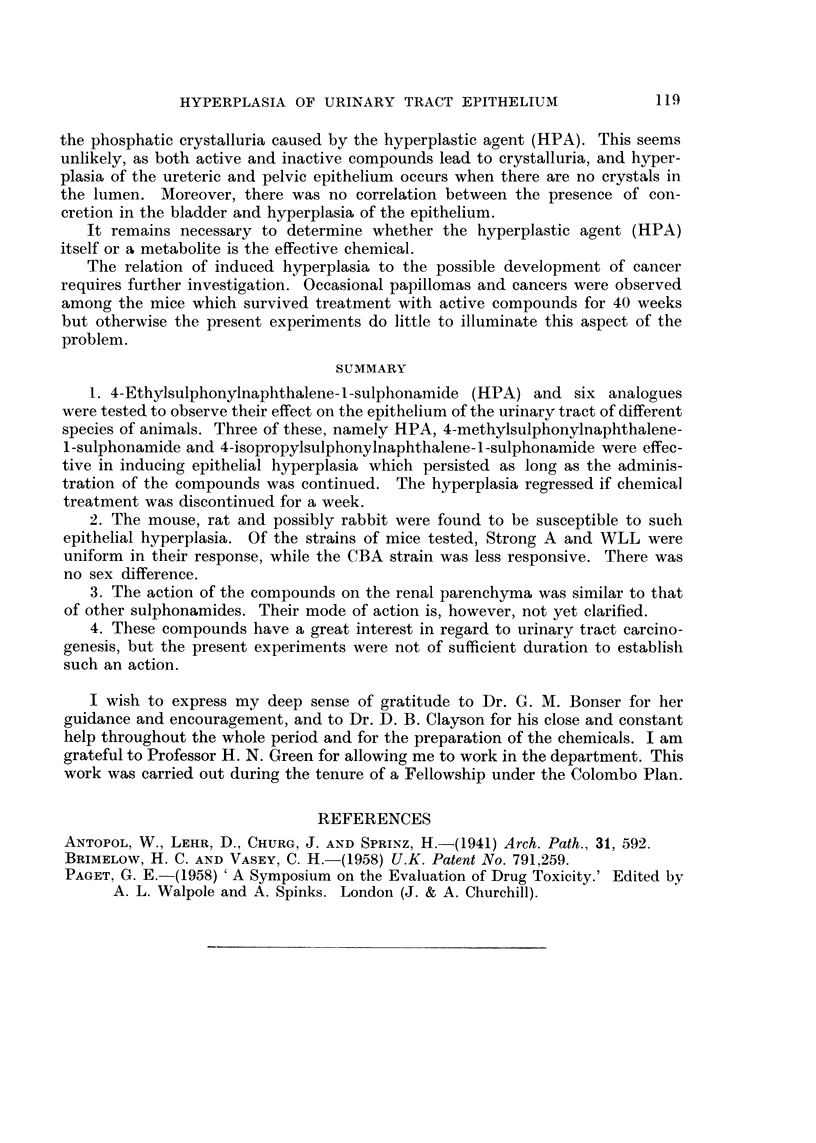# Hyperplasia of Urinary Tract Epithelium Induced by Continuous Administration of Sulphonamide Derivatives

**DOI:** 10.1038/bjc.1962.11

**Published:** 1962-03

**Authors:** K. P. Sen Gupta

## Abstract

**Images:**


### HYPERPLASIA OF URINARY TRACT EPITHELIUM INDUCED BY CONTINUOUS ADMINISTRATION OF SULPHONAMIDE DERIVATIVES

K. P. SEN GUPTA

*From the Department of Experimental Pathology and Cancer Research, University of Leeds, School of Medicine, Leeds, 2**

Received for publication November 3, 1961

PAGET (1958) AND WALPOLE (personal communication) found that the administration of 4-ethylsulphonylnaphthalene-1-sulphonamide (HPA) to rats of their closed random-bred Wistar colony induced hyperplasia of the urinary tract epithelium. The mechanism of its production was not elucidated although it was thought that the phosphatic crystalluria, which resulted from the ingestion of the chemical, might be involved. These workers obtained marked hyperplasia by the daily administration of 30, 60 or 120 mg./kg. body weight five times a week for 8 weeks, but found that as little as 0·01 per cent of the chemical in the diet (approximately 1 mg. daily) produced crystalluria and some hyperplasia.

This paper deals with the effects of HPA in various species, the effect of length of exposure on the degree of hyperplasia and whether or not the hyperplasia is reversible. The action of six similar compounds on the urinary tract epithelium of mice has also been examined.

### MATERIAL AND METHODS

*Chemicals.*—4-ethylsulphonylnaphthalene-1-sulphonamide (HPA) was prepared by Dr. D. B. Clayson, by the method of Brimelow and Vasey (1958). This chemical will be referred to as the hyperplastic agent or HPA. Six related chemicals were also prepared by the same method. Their chemical name, structure, melting point and abbreviation are given in Table I.

### *Experimental animals*

*Mice* of three inbred strains, 8–12 weeks old, were used for feeding experiments. They were white label (Leeds) or WLL, CBA and Strong A. All three strains were employed for testing HPA and H6, Strong A and WLL for H1, H3 and H4, and Strong A only for H2 and H5.

*Rats* belonged to a closed colony of albinos husbanded in Sheffield and originally derived from Wistar stock. Each rat weighed 200–250 g. at the start.

*Rabbits* belonged to a mixed stock bred in the laboratory. They weighed $4\frac{1}{2}$–6 lb. at the start.

*Guinea-pigs* were obtained from a local dealer or from the laboratory stock, and weighed 250–400 g. at the start.

* Permanent address: Department of Pathology and Bacteriology, Institute of Postgraduate Medical Education and Research, Calcutta, 20, India.

TABLE I.—*Description of the Chemical Compounds Under Investigation*

| Chemical name | Structure | Melting point | Abbreviation |
|---|---|---|---|
| 4-ethylsulphonylnaphthalene-1-sulphonamide |  | 198·0 | HPA |
| 4-isoprophylsulphonylnaphthalene-1-sulphonamide |  | 198·5–200·5 | H1 |
| 4-*n*-propylsulphonylnaphthalene-1-sulphonamide |  | 189·0–190·0 | H2 |
| 4-methylsulphonylnaphthalene-1-sulphonamide |  | 140·0–141·0 | H3 |
| N-methyl-4-ethylsulphonylnaphthalene-1-sulphonamide |  | 135·0–136·0 | H4 |
| N-piperidyl-4-ethylsulphonylnaphthalene-1-sulphonamide |  | 101·0–102·0 | H5 |
| 4-ethylsulphonylbenzene-1-sulphonamide |  | 184·0–185·0 | H6 |

*Dogs* were mongrels bought from a dealer, and weighed 18½ and 22 lb. at the start.

All animals received food and water *ad lib.*

*Methods of administration and doses of the chemicals in different animals*

*Mice.*—The chemicals were incorporated in the diet in a concentration of 100 mg./kg. (0·01 per cent). Powdered rat cake (obtained from the Northern Agricultural Society, Aberdeen) was mixed thoroughly with an acetone solution of the chemical and made into a thick paste with water. This paste was subsequently dried in a hot air oven at 56° C. for 48 hours.

*Rats.*—In the first trial the chemical (100 mg.) was added to the diet (1 kg.) in the usual way. Thereafter, the chemical was made into a thick suspension of 40 mg./ml. of arachis oil. 0·2 ml. of the suspension (8 mg. chemical) was given by stomach tube under light ether anaesthesia to each rat six times a week for 15 weeks.

*Rabbits.*—0·5 ml. of a suspension of 100 mg. chemical/ml. arachis oil (50 mg. chemical) was given by stomach tube under light ether anaesthesia six times a week for 15 weeks.

*Guinea-pigs.*—The suspension of the chemical was made in the same concentration as for rats but the dose was 20 mg. in 0·5 ml. oil, which was given by stomach tube under light ether anaesthesia six times a week for 15 weeks.

*Dogs.*—The chemical was given by mouth in powder form in a gelatin capsule. In one dog the dose was 120 mg. per day six times a week for 5 weeks. This dose was similar to that in mice in relation to the body weight. In the other dog, the dose was doubled; this animal received 250 mg./day six times a week for 4 weeks, followed by 500 mg./day six times for another week before it was killed.

*Technique of post mortem examination*

*Mice* were killed by ether. The urinary bladder was distended by injecting Bouin's fluid *per urethram.* The bladder, kidneys and ureters were dissected out *en bloc* and fixed intact on a thin sheet of cork. The latter was put in a large volume of fixative for 24 hours. The bladder was bisected vertically at right angles to the sagittal plane in order to obtain the intravesical portion of one or both ureters in the section. The kidneys were bisected in a plane which allowed the renal pelvis and upper end of the ureter to be included in the section.

*Rats, rabbits, guinea-pigs and dogs* were killed by ether, except the dogs, which received intravenous nembutal. The urinary bladder was distended with formol saline and the neck of the bladder tied by cotton thread. The kidneys with attached ureters were dissected out, fixed on a thin sheet of cork and put in a large volume of fixative. Sections were cut from different portions of the bladder, the ureteric orifice of the bladder, the mid portion of the ureters and from the renal pelvis and renal parenchyma. All the sections were stained by haematoxylin and eosin and, when required, by the periodic acid-Schiff method, Alcian blue and von Kossa's method for calcium.

### RESULTS

*Experiment 1—Testing of HPA and H3 in different strains of mice*

After a preliminary trial, which showed that HPA did induce urinary tract epithelial hyperplasia in the mouse, an experiment was planned as shown in Table II. In both male and female mice of the strains WLL and Strong A, there was urinary tract epithelial hyperplasia and the effect could be demonstrated as early as 5 weeks after continuous administration of the chemicals. The effect persisted, and became more advanced as the duration of the treatment increased. A few CBA mice appeared to respond in the early weeks, but not in the later part of the experiment. The impression was gained that H3 was rather more active than HPA.

TABLE II.—*Continuous Treatment of Mice with HPA and H3*

| Chemical | Strain | Number at start M | F | Number dying during experiment M | F | Number killed (weeks)* 5 | 10 | 15 | 20 | 25 | 30 | 35 | 40 | Hyperplasia of urinary tract epithelium Bladder | Ureter | Pelvis |
|---|---|---|---|---|---|---|---|---|---|---|---|---|---|---|---|---|
| HPA | WLL | 15 | 8 | 3 | 2 | 2 | 2 | 2 | 2 | 2 | 2 | 3 | 3 | + | + | + |
| | Strong A | 14 | 14 | 2 | 5 | 2 | 2 | 2 | 2 | 3 | 3 | 3 | 4 | + | + | + |
| | CBA | 20 | 8 | 6 | 1 | 2 | 2 | 2 | 2 | 3 | 3 | 3 | 4 | ± | ± | ± |
| H3 | WLL | 12 | 8 | 2 | 1 | 2 | 2 | 2 | 2 | 2 | 2 | 2 | 2 | + | + | + |
| | Strong A | 16 | 8 | 3 | 5 | 2 | 2 | 2 | 2 | 2 | 2 | 2 | 2 | + | + | + |

* Animals dying spontaneously not included.
\+ = Present.
± = Initial response not maintained.

A preliminary trial in a few mice had shown that the hyperplasia was reversible if the treatment was discontinued up to 10 weeks. In order to study this further, in addition to the mice shown in Table II, two mice were removed from the experiment at 20, 25, 30, 35 and 40 weeks and were given a normal diet for 7 days. In the HPA experiment this was done in all three strains ; with H3 it was done only in Strong A mice, except that 2 mice of the WLL strain were removed at 35 and 40 weeks. In all the mice thus treated, the hyperplasia subsided within one week following the cessation of treatment, but when organic change such as hydronephrosis or hydro-ureter was present this persisted.

TABLE III.—*Treatment of Rats, Rabbits and Guinea-pigs with HPA and H3*

| Chemical | Species | Number at start M | F | Daily dose | Methods of administration | Number dying during experiment | Number killed (weeks)* 2 | 10 | 15 | Hyperplasia of urinary tract epithelium Bladder | Ureter | Renal pelvis |
|---|---|---|---|---|---|---|---|---|---|---|---|---|
| HPA | Rat | 6 | 0 | 0·01% in diet | Daily in diet | 0 | 2 | 2 | 2 | — | — | — |
| | | 6 | 0 | 8 mg. | By stomach tube ×6 per week | 0 | 2 | 2 | 2 | + | + | — |
| | Rabbit | 2 | 4 | 50 mg. | | 2 | 1 | 1 | 2 | — | — | ? |
| | Guinea-pig | 3 | 3 | 20 mg. | | 2 | 1 | 1 | 2 | — | — | — |
| H3 | Rat | 6 | 0 | 0·01% in diet | Daily in diet | 0 | 2 | 2 | 2 | — | — | — |
| | | 6 | 0 | 8 mg. | By stomach tube ×6 per week | 1 | 1 | 2 | 2 | + | + | — |
| | Rabbit | 3 | 3 | 50 mg. | | 3 | 1 | 1 | 1 | — | — | ? |
| | Guinea-pig | 4 | 2 | 20 mg. | | 2 | 1 | 1 | 2 | — | — | — |

* Animals dying spontaneously not included.

*Experiment II—Testing of HPA and H3 in rats, rabbits, guinea-pigs and dogs (Table III)*

The trial dose of 0·01 per cent in the diet was ineffective in rats, but when it was increased to 8 mg. six times per week (i.e. seven-fold) for periods from 2–15 weeks, a positive response was obtained with both compounds. The response in the rabbit was doubtful with the dose used and guinea-pigs were refractory.

In addition, HPA only was tested in the dog for a period of 5 weeks. In the first dog, the dose was calculated to be proportionate to that used for mice. When this was ineffective, the dose was doubled for 4 weeks and quadrupled for one week. Even so, no hyperplasia was obtained. The results are summarised in Table IV.

TABLE IV.—*Summary of Continuous Treatment with HPA in Different Species for Varying Lengths of Time*

| Species | Number at start | Period of observation (weeks) | Hyperplasia of urinary tract epithelium: Bladder | Ureter | Renal pelvis |
|---|---|---|---|---|---|
| Mice | 79 | 5–40 | + | + | + |
| Rats | 12 | 2–15 | + | + | — |
| Rabbits | 6 | 2–15 | — | — | ? |
| Guinea-pigs | 6 | 2–15 | — | — | — |
| Dogs | 2 | 5 | — | — | — |

*Experiment III—Testing of H*1, *H*2, *H*4, *H*5 *and H*6

These compounds were tested on mice of one or both of the strains WLL and Strong A in the dose found to be effective for HPA and H3 (Table V). H2, H4,

TABLE V.—*Treatment of Different Strains of Mice with H*1, *H*2, *H*4, *H*5 *and H*6 *in a Dose of* 0·01 *per cent in Diet*

| Chemical | Strain | Number at start: M | F | Number dying during experiment: M | F | Number killed (weeks)*: 5 | 10 | 15 | 20 | 25 | 30 | 35 | 40 | Hyperplasia of urinary tract epithelium: Bladder | Ureter | Renal pelvis |
|---|---|---|---|---|---|---|---|---|---|---|---|---|---|---|---|---|
| H1 | WLL | 4 | 9 | 1 | 1 | 2 | 2 | 2 | 2 | 2 | 1 | .. | .. | + | + | + |
| | Strong A | 14 | 10 | 1 | 2 | 2 | 2 | 2 | 2 | 2 | 3 | 3 | 4 | + | + | + |
| H2 | Strong A | 13 | 14 | 6 | 5 | 2 | 2 | 2 | 2 | 2 | 2 | 2 | 2 | — | — | — |
| H4 | WLL | 7 | 0 | 1 | 0 | 2 | 2 | .. | 2 | .. | .. | .. | .. | — | — | — |
| | Strong A | 14 | 10 | 4 | 3 | 2 | 2 | 2 | 2 | 2 | 2 | 2 | 3 | — | — | — |
| H5 | Strong A | 6 | 0 | 0 | 0 | 2 | 2 | 2 | .. | .. | .. | .. | .. | — | — | — |
| H6 | WLL | 11 | 6 | 2 | 1 | 2 | .. | 2 | 2 | 2 | 2 | 2 | 2 | — | — | — |
| | Strong A | 27 | 10 | 10 | 1 | 2 | 2 | 2 | 2 | 2 | 2 | 2 | 2 | — | — | — |
| | CBA | 15 | 9 | 9 | 5 | 2 | 2 | 2 | .. | .. | .. | 2 | 2 | — | — | — |

* Animals dying spontaneously not included.
\+ = Present.
— = Absent.

H5 and H6 were completely inactive, but it is to be noted that as H5 was available only in small quantity, the test was not continued beyond 15 weeks. H6 was also tested in CBA mice. The mortality was high, but in 6 mice killed between 5 and 15 weeks and 4 at 35–40 weeks no hyperplasia was observed.

*The structure of the normal urinary tract epithelium of the mouse*

The bladder is lined by transitional epithelium 2–3 layers thick. The cells of the basal layer tend to be vacuolated, while the cytoplasm of the superficial layer is faintly eosinophilic and granular. The funnel-shaped renal pelvis surrounds the single papilla. Near the fornix it is lined with stratified transitional epithelium, which becomes thicker (4–5 layers) as the entrance to the ureter is reached. The renal papilla fills almost the whole of the pelvis and is covered with a single layer of cuboidal epithelium (Fig. 1).

The ureter is lined by transitional cell epithelium 3 to 4 layers thick. The basal cells are vacuolated, while the superficial cells have faintly granular eosinophilic cytoplasm. When the ureter is partially contracted, even the folded eipthelium has a similar appearance (Fig. 2).

### *Changes in the Urinary Bladder, Kidney and Ureter in the Mouse and Rat Following Continuous Administration of the Active Chemicals*

#### *Macroscopical*

*Urinary bladder*

In the rat, a few fine crystals were seen inside the bladder when the higher doses were employed. In the mouse, tiny white crystals were frequently found. In some cases, large multifaceted stones were present and caused distension of the organ. There was no correlation between the presence of the stone, large or small, and hydronephrosis, which was frequently unilateral rather than bilateral. Moreover, similar crystals or stones were equally frequent in the bladders of the mice treated with the inactive compounds.

*Renal parenchyma*

In both the rat and the mouse, the gross changes could be divided into two main types: (i) hydronephrosis of either early pelvic or late severe type, and (ii) scarred and/or contracted kidneys.

(i) *Hydronephrosis.*—In the rats treated with HPA and H3 there was only one instance of right-sided pelvic type of hydronephrosis at the end of 15 weeks. In the mouse, unilateral hydronephrosis was often observed even in the early stages of administration of the chemicals and as early as 5 weeks after the start of treatment. Where the hydronephrosis was bilateral, the right kidney was usually more severely affected than the left. The severe type of hydronephrosis was less frequent and was observed only in the late stages of the experiments. With inactive chemicals these changes were infrequent.

(ii) *Scarred and/or contracted kidneys.*—Scarring of a portion of one or both kidneys was rarely observed before 10–15 weeks. Sometimes the whole kidney was reduced in size and had a finely granular surface. The renal and pelvic changes were observed simultaneously in some instances, causing difficulty in classifying the lesions, which were then recorded under the predominant lesion.

(iii) *Ureter.*—The upper portion of the ureter was usually dilated if the pelvic type of hydronephrosis was present. Sometimes the whole ureter was affected on one or both sides. Stones were not found in the ureter.

The results of the macroscopical observations are summarised in Table VI.

#### *Microscopical*

*Urinary bladder*

The changes in the urinary bladder of the rat and the mouse could be subdivided into three varieties :

(i) *Focal epithelial hyperplasia.*—This usually affected different areas of the same bladder and appeared to be the earliest change following administration of

TABLE VI.—*Macroscopical Appearance of the Urinary Bladder, Renal Parenchyma and Ureter in Mice after Continuous Treatment with HPA, H3 and H1*

| Chemical | Strain | Number at start | | Number dying during experiment | | Urinary bladder | | State of kidneys | | | | Ureters | |
|---|---|---|---|---|---|---|---|---|---|---|---|---|---|
| | | | | | | | | | Hydronephrosis | | Scarred and/or contracted | | |
| | | M | F | M | F | Normal | Concretion | Normal | Pelvic | Severe | | Normal | Dilated |
| HPA | . Strong A | 14 | 14 | 2 | 5 | 9 | 12 | 14 | 16 | 6 | 6 | 26 | 16 |
| | WLL | 15 | 8 | 3 | 2 | 8 | 10 | 16 | 10 | 2 | 8 | 25 | 11 |
| H3 | . Strong A | 16 | 8 | 3 | 5 | 6 | 10 | 14 | 8 | 4 | 6 | 20 | 12 |
| | WLL | 12 | 8 | 3 | 1 | 7 | 9 | 16 | 8 | 3 | 5 | 22 | 10 |
| H1 | . Strong A | 14 | 10 | 1 | 2 | 10 | 11 | 16 | 11 | 8 | 7 | 23 | 19 |
| | WLL | 4 | 9 | 1 | 1 | 3 | 8 | 8 | 6 | 3 | 5 | 14 | 8 |

### EXPLANATION OF PLATES

FIG. 1.—Normal mouse renal pelvis. The papilla is covered by a single layer of cuboidal epithelium. The parietal wall of the pelvis is lined by stratified transitional epithelium which becomes thicker as the top of the ureter is approached. ×45.

FIG. 2.—Mid portion of normal mouse ureter. The basal cells are vacuolated while the superficial cells are finely granular. ×65.

FIG. 3.—In this and the succeeding figures the bladder epithelium is uppermost. Urinary bladder from Strong A mouse after 5 weeks of HPA in diet. There is transition from normal epithelium on the right to an area of focal hyperplasia several layers thick on the left. There is slight oedema of the subepithelial tissues and scanty cellular infiltration. The muscle is normal. ×45.

FIG. 4.—Mouse bladder after 15 weeks of HPA in diet. The whole epithelium is 5–8 layers thick. There is oedema of the subepithelial tissues. ×45.

FIG. 5.—Mouse bladder after 20 weeks of HPA in diet. There are two papillary projections of epithelium several layers thick, one with and the other without a core. These are part of a general hyperplasia (well seen on the left) and are not tumours. ×100.

FIG. 6.—Bladder from rat after 15 weeks of HPA by stomach tube, showing diffuse epithelial hyperplasia leading to folding, and slight subepithelial oedema. ×33.

FIG. 7.—Mouse bladder showing diffuse hyperplasia, 5–6 layers thick. Nuclei are hyperchromatic and there are bizarre nuclei and occasional mitoses. ×125.

FIG. 8.—Mouse bladder after 40 weeks of H1 in diet, showing transitional cell papilloma with formation of lumina. ×20.

FIG. 9.—Mouse bladder after 40 weeks of H3 in diet, showing extensive transitional cell carcinoma. ×40.

FIG. 10.—Rat kidney after 15 weeks of HPA by stomach tube. There is a cast of calcium salts in a renal tubule in the centre, with partial necrosis of the epithelial cells. There is also amorphous deposit in the adjacent tubule below. ×125.

FIG. 11.—Kidney from Strong A mouse after 20 weeks of HPA in diet. There is a laminated cast of calcium salts in the tubule near the space (above) and also a collection of lymphocytes in the adjoining area. ×125.

FIG. 12.—Mouse kidney after 20 weeks of HPA in diet, showing cortex to left and medulla to right. There is widespread deposition of calcium salts, which appear black, in the lumen of both cortical and medullary tubules. ×20.

FIG. 13.—Section of whole mouse kidney after 15 weeks of HPA in diet. Pelvic type of hydronephrosis with compression of the papilla and general dilatation of the tubules. There is marked epithelial hyperplasia of the renal pelvis. ×12.

FIG. 14.—High power view of the same renal pelvis as in Fig. 13. There is uniform epithelial hyperplasia 6–8 layers thick at the entrance to the ureter. ×45.

FIG. 15.—Mouse bladder near the orifice of the ureter. The ureteric epithelium shows marked hyperplasia causing a slit-like lumen, while the epithelium of the bladder (on left) shows diffuse hyperplasia and there is also oedema of the subepithelial tissues. ×33.

FIG. 16.—Dilated ureter from a mouse after 20 weeks of HPA, at the same magnification as Fig. 2. There is uniform epithelial hyperplasia with desquamated epithelial cells within the lumen and gross dilatation of the ureter. ×65.

1

2

3

4

5

6

Sen Gupta.

7

8

9

10

11

12

13

14

15

16

Sen Gupta.

the chemicals. In the preliminary trial, initial necrosis of the epithelium was observed after two weeks, and focal hyperplasia as early as 3 or 4 weeks. The latter was, however, more uniformly observed 5 weeks following the start of treatment. Transition could be seen from a normal epithelium of 2 or 3 layers thick to an area of 5 or 6 layers thick in a well distended bladder. The subepithelial tissues usually showed some oedema with scanty cellular infiltration (Fig. 3). In some instances, severe inflammation affected the entire bladder wall or focal areas of lymphocytic infiltration were seen in the subepithelial tissues. There was no correlation between hyperplasia of the epithelium and the presence of inflammation. Indeed with H4, severe inflammation of the bladder wall was often observed without any evidence of consistent hyperplasia.

(ii) *Diffuse epithelial hyperplasia.*—This affected the entire urinary bladder, the epithelium being 6 or 8 layers thick. It was associated with oedema of the subepithelial tissues (Fig. 4). In more severe lesions, there were papillary excrescences of the increased layers either with or without a core (Fig. 5). The basal cells sometimes grew into the subepithelium due to folding (Fig. 6). The nuclei were hyperchromatic (Fig. 7) and occasional mitoses were observed. These changes were seen in rats after 15 weeks and mice after 20 weeks and persisted as long as the chemicals were administered. There was, however, no correlation between the degree of hyperplasia and the length of the administration of the chemical.

(iii) *Papilloma and carcinoma.* There were two papillomas with H1 and one carcinoma with H3 after 40 weeks of continuous administration. The papillomas were transitional cell in type with the formation of acini (Fig. 8). One papilloma was situated on the lateral wall between the dome and the neck and the other was at the dome of the bladder. Carcinoma affecting two areas in the same bladder was present in one mouse treated with H3. This was also transitional cell in type with infiltration of the subepithelial tissue as far as the muscle layer but not invading it (Fig. 9).

*Renal parenchyma*

There were three types of change in the renal parenchyma in the rat and mouse.

(i) *Tubular degeneration and/or deposition of calcium.*—These changes occurred either simultaneously or separately. Deposits of globular amorphous calcium were seen in the convoluted and distal tubules, either in the early or late stages of treatment. When these occluded the lumen, there was concomitant necrosis of the epithelial cells with subsequent regeneration. Some of these deposits appeared laminated (Fig. 10 and 11). Similar depositions of calcium salts were observed in the renal papilla and led to necrosis of the overlying epithelium. Subsequent regeneration could be seen around such areas. In some instances calcium deposition was remarkably heavy in the corticomedullary areas but in others it was irregularly distributed in the parenchyma (Fig. 12).

(ii) *Pyelonephritis.*—The deposition of calcium salts in the tubule seemed to initiate an interstitial inflammation. This was focal or diffuse, affecting simultaneously different parts of the same kidney and resulting in scarred or small contracted kidneys. Sometimes a scarred kidney was observed without any deposition of calcium salts but usually associated with hydronephrosis.

(iii) *Hydronephrotic atrophy.*—In the early pelvic type of hydronephrosis, the parenchymal change consisted of focal dilatation of the collecting tubules, but sometimes other parts of the nephron were affected. Later on, the renal papilla was gradually compressed and more generalised tubular dilatation of both cortex and medulla took place (Fig. 13). In this stage frequently there was associated interstitial inflammation especially in mice treated for 15 weeks and more.

*Renal pelvis and ureter*

In some mice epithelial hyperplasia 7–8 layers thick occurred at the upper end of the ureter and caused the early pelvic type of hydronephrosis (Fig. 14). In others, this hyperplasia affected the whole ureter, resulting in narrowing of the lumen into a thin slit (Fig. 15). Similar hyperplasia was seen even in dilated ureters (Fig. 16).

### DISCUSSION

The initial observation of Walpole (personal communication) and Paget (1958) that 4-ethylsulphonylnaphthalene-1-sulphonamide (HPA) induces hyperplasia of urinary tract epithelium has been confirmed. In the present experiments it was shown that hyperplasia was caused by repeated doses of an amount of chemical which the animals tolerated well and that dosing had to be continued until the animals were killed. The hyperplasia subsided if an interval of one week occurred between the cessation of the treatment and death. The epithelium of the bladder was more uniformly affected than that of either the renal pelvis or ureter, but if the changes were present in two or more sites, they seemed to arise concomitantly rather than by spread from the bladder through the ureter to the pelvis. The hyperplasia could be the primary and only change observed, or it could follow initial necrosis or be associated with subepithelial oedema and inflammation. In the kidney parenchyma, the changes were similar to those observed with other sulphonamides, namely, tubular degeneration and calcification with an intense inflammatory reaction (Antopol *et al.*, 1941).

The hyperplastic effect was most apparent in the mouse, but the rat and possibly the rabbit were also affected. No response was observed in the guinea-pig or, in experiments on only two animals, in dogs. Mice of the Strong A and white label (Leeds) strains were equally affected, whereas those of the CBA strain were less sensitive, the renal tract epithelium undergoing mild hyperplastic changes only, which did not become more intense even when treatment was continued for 40 weeks. The sexes were equally affected in all cases.

Six analogues of the hyperplastic agent (HPA) were tested, of which two were active, namely 4-methylsulphonylnaphthalene-1-sulphonamide (H3) and 4-isopropylsulphonylnaphthalene-1-sulphonamide (H1). It is apparent that comparatively minor alterations in the chemical structure of the hyperplastic agent (HPA) are sufficient to suppress its activity. From the limited number of compounds tested it appears that an unsubstituted sulphonamide group is necessary for activity, because both the monomethyl and the piperidyl-substituted sulphonamides were inactive. Similarly, the observation that 4-*n*-propylsulphonyl-naphthalene-1-sulphonamide was inactive, whereas the isopropyl derivative was active, indicates that the sulphonyl part of the molecule is also important.

The mechanism of action of these chemicals requires further study. Walpole and Paget were of the opinion that the hyperplasia might have been induced by the phosphatic crystalluria caused by the hyperplastic agent (HPA). This seems unlikely, as both active and inactive compounds lead to crystalluria, and hyperplasia of the ureteric and pelvic epithelium occurs when there are no crystals in the lumen. Moreover, there was no correlation between the presence of concretion in the bladder and hyperplasia of the epithelium.

It remains necessary to determine whether the hyperplastic agent (HPA) itself or a metabolite is the effective chemical.

The relation of induced hyperplasia to the possible development of cancer requires further investigation. Occasional papillomas and cancers were observed among the mice which survived treatment with active compounds for 40 weeks but otherwise the present experiments do little to illuminate this aspect of the problem.

### SUMMARY

1. 4-Ethylsulphonylnaphthalene-1-sulphonamide (HPA) and six analogues were tested to observe their effect on the epithelium of the urinary tract of different species of animals. Three of these, namely HPA, 4-methylsulphonylnaphthalene-1-sulphonamide and 4-isopropylsulphonylnaphthalene-1-sulphonamide were effective in inducing epithelial hyperplasia which persisted as long as the administration of the compounds was continued. The hyperplasia regressed if chemical treatment was discontinued for a week.

2. The mouse, rat and possibly rabbit were found to be susceptible to such epithelial hyperplasia. Of the strains of mice tested, Strong A and WLL were uniform in their response, while the CBA strain was less responsive. There was no sex difference.

3. The action of the compounds on the renal parenchyma was similar to that of other sulphonamides. Their mode of action is, however, not yet clarified.

4. These compounds have a great interest in regard to urinary tract carcinogenesis, but the present experiments were not of sufficient duration to establish such an action.

I wish to express my deep sense of gratitude to Dr. G. M. Bonser for her guidance and encouragement, and to Dr. D. B. Clayson for his close and constant help throughout the whole period and for the preparation of the chemicals. I am grateful to Professor H. N. Green for allowing me to work in the department. This work was carried out during the tenure of a Fellowship under the Colombo Plan.

### REFERENCES

Antopol, W., Lehr, D., Churg, J. and Sprinz, H.—(1941) *Arch. Path.*, **31**, 592.

Brimelow, H. C. and Vasey, C. H.—(1958) *U.K. Patent No.* 791,259.

Paget, G. E.—(1958) ' A Symposium on the Evaluation of Drug Toxicity.' Edited by A. L. Walpole and A. Spinks. London (J. & A. Churchill).